# A Brief History of the Major Rickettsioses in the Asia–Australia–Pacific Region: A Capstone Review for the Special Issue of *TMID*

**DOI:** 10.3390/tropicalmed5040165

**Published:** 2020-10-27

**Authors:** Daniel H. Paris, Daryl J. Kelly, Paul A. Fuerst, Nicholas P. J. Day, Allen L. Richards

**Affiliations:** 1 Department of Medicine, Swiss Tropical and Public Health Institute, 4051 Basel, Switzerland; 2 Department of Clinical Research, University of Basel, 4051 Basel, Switzerland; 3 Department of Evolution, Ecology and Organismal Biology, The Ohio State University, Columbus, OH 43210, USA; kelly.350@osu.edu (D.J.K.); fuerst.1@osu.edu (P.A.F.); 4 Mahidol-Oxford Tropical Medicine Research Programme, Faculty of Tropical Medicine, Mahidol University, 420/6 Rajvithee Road, Bangkok 10400, Thailand; nickd@tropmedres.ac; 5 Center for Tropical Medicine, Nuffield Department of Clinical Medicine, Churchill Hospital, Old Road, Headington, Oxford OX3 7LJ, UK; 6 Department of Preventive Medicine and Biostatistics, Uniformed Services University of the Health Sciences, Bethesda, MD 20814, USA; Allen.Richards@comcast.net

**Keywords:** typhus, epidemic typhus, murine typhus, scrub typhus, spotted fever rickettsiae, *Orientia*, *O. tsutsugamushi*, *R. typhi*, genome comparison, diagnostic tools

## Abstract

The rickettsioses of the “Far East” or Asia–Australia–Pacific region include but are not limited to endemic typhus, scrub typhus, and more recently, tick typhus or spotted fever. These diseases embody the diversity of rickettsial disease worldwide and allow us to interconnect the various contributions to this special issue of Tropical Medicine and Infectious Disease. The impact of rickettsial diseases—particularly of scrub typhus—was substantial during the wars and “police actions” of the last 80 years. However, the post-World War II arrival of effective antibiotics reduced their impact, when recognized and adequately treated (chloramphenicol and tetracyclines). Presently, however, scrub typhus appears to be emerging and spreading into regions not previously reported. Better diagnostics, or higher population mobility, change in antimicrobial policies, even global warming, have been proposed as possible culprits of this phenomenon. Further, sporadic reports of possible antibiotic resistance have received the attention of clinicians and epidemiologists, raising interest in developing and testing novel diagnostics to facilitate medical diagnosis. We present a brief history of rickettsial diseases, their relative importance within the region, focusing on the so-called “tsutsugamushi triangle”, the past and present impact of these diseases within the region, and indicate how historically, these often-confused diseases were ingeniously distinguished from each another. Moreover, we will discuss the importance of DNA-sequencing efforts for *Orientia tsutsugamushi*, obtained from patient blood, vector chiggers, and rodent reservoirs, particularly for the dominant 56-kD type-specific antigen gene (*tsa56*), and whole-genome sequences, which are increasing our knowledge of the diversity of this unique agent. We explore and discuss the potential of sequencing and other effective tools to geographically trace rickettsial disease agents, and develop control strategies to better mitigate the rickettsioses.

## 1. Introduction

Of all infectious diseases the world has experienced, few have truly altered human history. In centuries past, diseases such as smallpox, plague, tuberculosis, and epidemic typhus (aka, louse-borne typhus) have been responsible for millions of deaths through uncontrolled outbreaks. The military impact has been dramatic. Hans Zinsser (1878–1940), bacteriologist and immunologist, famous for his significant work on typhus, stated that the explosive outbreaks of epidemic typhus influenced the outcome of more wars between the 16th and the 19th century than any soldier or general [1]. Now in the 21st century, many of these diseases, including the rickettsial diseases that we will explore here, or have been examined by others in this special issue of *Tropical Medicine and Infectious Disease* (TMID), remain of international public health concern [2,3].

The discovery of antimicrobial agents in the last century altered human perceptions of epidemics of infectious diseases and the associated effects of large outbreaks. Our wartime experiences of the past 100 years led to improved recognition and treatments for many infectious diseases not previously defined. Ironically, the interest in these diseases rapidly declined when effective antimicrobials or vaccines became available and the once devastating diseases became controllable, even though wars themselves subside. This poses a problem for endemic areas, where the diseases may persist. Despite available treatment, the lack of clinical awareness, often coupled with diagnostic difficulties, can lead to under-recognition with considerable morbidity and preventable mortality. The history of “typhus” in the Far East and the Asia–Australia–Pacific region follows this pattern. The broad topic for this TMID special issue, “*The Past and Present Threat of Rickettsial Diseases*”, leads us to focus on one region of the world that is hyperendemic, both historically and presently, for a host of rickettsial diseases. This is a vast area we are calling the “Asia–Australia–Pacific” or AAP region. For a map of the region, see the paper in this special issue by Luce-Federow et al. [4]. Indeed, the majority of individual papers that appear in this TMID special issue describe rickettsioses or rickettsial pathogens endemic to the AAP region.

Today the reported incidences of rickettsioses in the world and for our purposes within the AAP are increasing. This is especially true of scrub typhus (rural, chigger-borne or mite-borne typhus), and to a lesser extent murine typhus (endemic, urban, flea-borne, or shop typhus) and spotted fever. Scrub and murine typhus represent the two most common forms of rickettsial diseases in these regions. Other rickettsioses such as tick-borne rickettsioses and spotted fever, though relatively infrequent in the AAP, are, nevertheless, endemic in the region [5]. In tropical regions, epidemic typhus occurs only rarely. Improved recognition of the disease syndromes by clinicians within the endemic regions and improved more accurate, easy-to-use diagnostics have led in recent years to enhanced recognition. Thus, rickettsioses such as scrub typhus are now being reported within an ever-growing, geographically disparate region [6,7]. In contrast, epidemic typhus caused primarily by *Rickettsia prowazekii*-infected lice (*Pediculus humanus corporis*) has played a relatively minor role in modern times as a cause of fevers in the AAP. During the Korean conflict (1950–1953), epidemic typhus had a significant impact on the civilian population. This was due, in part, to growing resistance of the vector body louse to dichlorodiphenyltrichloroethane (DDT) [3,8]. The low incidence of epidemic typhus in the AAP is not unexpected, given that most instances of epidemic typhus are associated with cold-weather areas and the infestation of cold-weather clothing by lice. Increases, or even limited outbreaks, of epidemic typhus have not been recently reported in the AAP. The vast majority of the rickettsioses reported within the AAP appear to be scrub typhus, caused by *Orientia tsutsugamushi*. Consistent with this finding, scrub typhus is part of the focus in 10 of the 19 papers within this special issue in which rickettsial diseases in the AAP are examined [4,6,9,10,11,12,13,14,15]. Specific instances in which scrub typhus in the AAP is a focus include Naoi et al. [13], who describe recent disparate clinical syndromes associated with scrub typhus in Japan. Gautam [16] reported scrub typhus cases in central Nepal, and recent fever studies suggest the presence of rickettsioses in mountainous regions of Bhutan [15].

The other rickettsioses, though relatively infrequent in the AAP, are, nevertheless, endemic in the region, and recent studies, including several in this special issue, suggest the influence of the other rickettsioses in the region [17,18,19]. The responsible agents (e.g., *Rickettsia honei, R. australis, R. japonica, R. felis*) do occur in the region, and some have been gaining traction. Most of these rickettsial agents are transmitted by ticks or fleas. For example, Australian cases of spotted fever group rickettsiosis (SFGR), or Queensland tick typhus (QTT), were reported in the 1940s with agent isolation from ticks in the early 1970s and were identified as *Rickettsia australis* [20]. In this special edition Stewart et al. [21] report the retrospective serodiagnosis of 36 recent cases of QTT occurring between 2000 and 2015. Also, in this special edition, Salgado Lynn et al. report a spotted fever group rickettsiosis in a wildlife researcher working in Sabah, Malaysia [22].

In northern Thailand, the Thai tick typhus agent, TT-118, now called *Rickettsia honei*, was initially recovered in 1962 from an ixodid tick near Chiang Mai [23], but the associated human rickettsiosis was not identified until 1994 [24]. A similar agent was also recovered from a patient on Flinders Island in South Eastern Australia in 1991 [25], and a molecular isolate was obtained from a patient of Bangkok, Thailand [26]. Another SFGR, named *Rickettsia japonica,* briefly described above, was isolated from the blood of a patient in Japan and found to cause febrile illness [27]. In this special issue of TMID on rickettsial diseases, Sando et al. [14] test the hypothesis that serological cross-reactivity of *O. tsutsugamushi* and *R. japonica* occurs in blood of infected patients—they found no cross-reaction. Tshokey, et al. [15] report the prevalence of all rickettsial groups and Q-fever group, in patients even in lightly explored mountainous regions of the AAP such as the historically remote south-central Asian country of Bhutan. Yuhana [28] deals with similar issues with murine typhus in Malaysia.

The occurrence of rare cases of epidemic typhus and sporadic occurrence of SFGR cases throughout the AAP, however, can be viewed in stark contrast to scrub typhus. Thus, we have chosen to place our primary focus on one disease in particular, scrub typhus, as it occurs in the AAP region. To understand best the status of scrub typhus in the AAP, it is useful to review the progression of diagnosis of rickettsial diseases.

Because research on disease in the AAP region has historically played a major role in redefining rickettsial diseases, we present here a brief account of the rickettsial diseases that have been lumped originally under the misleading term “tropical typhus” and consider their relative importance within the region, focusing on the so-called “tsutsugamushi triangle.” (For further consideration of the term “tropical typhus”, a 1983 lecture by George Lewis [29] explores the term). We describe the past and present impact of these diseases within the region, and present how these often-confused diseases were ingeniously distinguished from one another. Finally, we explore the potential to use genetic tools to trace geographically the spread of disease agents. Along with other factors, increasingly effective diagnostic tools and spread of expertise to use these tools should allow scientists and physicians to explain the spread of disease and thus initiate possible strategies to better control these rickettsioses. This review represents a capstone to the special issue, using the rickettsial diseases occurring in tropical Asia to highlight the continuing open questions, while indicating how the contributions to this issue have advanced our understanding of rickettsial diseases.

## 2. History of Typhus

### 2.1. Early Accounts and History of “Typhus”

It is impossible to define the “earliest account” of typhus since the term “typhus” was applied to a broad range of infectious diseases before the 20th century. Before scientific criteria for “typhus” were developed, the ancient term used was the Greek equivalent of “plague”. “Plague” comprised a collection of diseases (not necessarily of infectious origin) with epidemic character and often included bubonic pest, typhus, dysentery, yellow fever, cholera, meningococcal diseases, scurvy, syphilis, and importantly, variola (smallpox). The works of Hippocrates, 460 BC [30], allow insight into how a more specific term “typhus” (τῦφος) was applied to the confused states frequently associated with high fevers. Many historians believe that Thucydides described “classical typhus” (epidemic typhus, as it is known today) for the first time during the plague of Athens in 430–425 BC [31]. Hans Zinsser also noted that the first book of “*L’epidemion*”, by Hippocrates, contained descriptions of potential typhus cases [1]. Few manuscripts survived, and little is known about epidemics that followed the “Athens plague”, until the “pest of Carthago” around 253 AD, in which the Bishop Saint Cyprien describes the characteristics of typhus [32]. A variety of epidemic-like diseases afflicted military campaigns during the Crusades (first in 1095, eighth and last in 1270), among which typhus was regularly noted [1]. Medical historians hypothesize that typhus was introduced from the Orient and/or from Africa during the first century and reached Spain around the 12th–14th century, from where the Spanish explorers exported it to the Americas.

The earliest concise accounts consistent with classical typhus arise from the end of the 15^th^ century and underscore its later association with times of crisis, wars and famine [1]. The disease entity typhus is purported to have killed uncounted millions of people from the 16^th^ century on, and over a further three and a half centuries “typhus” was gradually redefined as a collection of distinctive diseases that affected specific populations [1]. A timeline of significant observations that have resulted in our current understanding of typhus-like disease is provided in Supplementary Table S1. By the mid–late 19th century, the term was gradually separated into a triad of diseases: typhus, typhoid, and relapsing fevers, based on clinical distinctions; exanthematic, enteric, and relapsing fevers [33,34]. The introduction of the descriptive term “*Typhus exanthématique”* by Boisier de Sauvages in Montpellier (1760) was an important attempt to distinguish the disease epidemic typhus [35].

As the confusion gradually lifted, the term typhus was increasingly associated with epidemic typhus fever, which over time received the appellation of “classic typhus”. However, the secrets of typhus were only unraveled a few years before World War I (WWI). The years from 1910 to 1915 proved to be important for rickettsia-associated typhus research, with a number of substantial discoveries made around the world to determine the causative agents, their vectors, and the identification of hosts and reservoirs. This resulted in the recognition of rickettsial diseases as distinct diseases and the creation of “Rickettsiology” as a discipline. The impact of this developing science on our appreciation of the impact of epidemic typhus in particular became pronounced. During the war and post war-time period between 1917 and 1923, 30 million cases and 3 million deaths were recorded in European Russia alone, where combatants were returning home [1].

### 2.2. The Confusion of “Typhus”

Investigations into typhus and its related forms were intense in Europe and America around WWI with the driving philosophy that typhus was contagious, epidemic and associated with overpopulation and poverty. Due to difficulties in culturing rickettsiae, the etiologic relationships between typhus and other similar diseases were not firmly established until the 1930s, but typhus fever was regarded as a unitary disease. Rocky Mountain spotted fever (RMSF) and Japanese spotted fever (JSF) were considered as belonging to different categories of disease, although they were known to be transmitted to humans by ticks, which may act as the reservoir of the agent, or which may acquire the agent from animals such as squirrels, chipmunks, rats, or related forms [19].

A new era of rickettsiology had been triggered by the discovery of an innovative diagnostic test for typhus by Weil and Felix in 1916 [36]. In 1915, during an epidemic typhus outbreak in Galicia (Spain), Weil and Felix isolated *Bacillus proteus*, a strain that caused agglutination when mixed with sera from typhus patients. Subsequent tests showed cross-reaction agglutination with whole antigen from this bacillus, subsequently reclassified in the genus *Proteus.* The *Proteus* species used was termed OX19 (X = undefined, later found to be *P. vulgaris)*.

In most geographic regions, the Weil–Felix test successfully identified epidemic typhus from other fevers. Increasingly, however, the original Weil–Felix test suggested complexity for typhus diagnosis in the tropics. Fletcher [37,38], in Malaya, found weak positivity in sporadic typhus and tsutsugamushi disease cases, but strong positivity for Brill’s disease (recrudescence of *R. prowazekii*) and a form of typhus from Australia, described by Hone in 1922 [39].

In the 1920s, an alternative strain of bacteria from the culture collection of the Bland-Sutton Institute at the Middlesex Hospital in London was brought to Southeast Asia to be used in the Weil–Felix array. This strain, defined as *B. proteus* X19, was actually a strain of *P. mirabilis* rather than *P. vulgaris*, and termed OXK (K = Kingsbury, who characterized it). The accidental use of the OXK strain subsequently became the cornerstone of “scrub typhus” diagnostics. From the background of “tropical typhus”, Fletcher in 1923 was the first to show that using the Weil–Felix test with antigens from X19 and OXK could subdivide “tropical typhus” [38,40]. For Indian tick typhus, the Weil–Felix reaction was usually negative or weakly positive to *Proteus* OX19, and negative for OXK, whereas Fletcher found two contrasting groups of cases of “tropical typhus”, one group which strongly agglutinated *Proteus* OX19 but not OXK and a second group showing the reverse. The mistake that allowed OX19 to be used for diagnosis resulted in Fletcher having the ability to distinguish two diagnostic groups associated with different epidemiologies. One group was associated with patients from rural jungle areas (hence “bush” or “scrub” typhus). This was similar to the Japanese tsutsugamushi disease, which had been considered until then to possibly represent a separate entity. The second group distinguished by Fletcher represented the discovery of the closely related “shop typhus” from urban areas (similar to Brill’s disease, later termed murine typhus, a flea-borne disease of Malaya [41]). The defined dilution titers, and characterized reference stains that were used, allowed a crude form of standardization. This enabled workers in different parts of the world to compare their findings.

**Figure 1:**
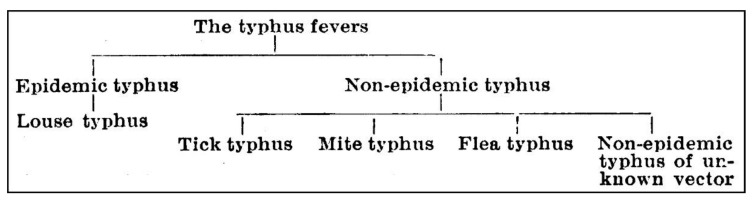
Megaw’s scheme identifying the forms of typhus present in India and Southeast Asia [46].

The work by Fletcher and Lewthwaite and many others at the Institute for Medical Research (IMR) in Kuala Lumpur triggered numerous reports from around the world describing diseases, which, while resembling epidemic typhus, were milder diseases that occurred sporadically. In the British colonies within the AAP, expatriate military personnel, especially in India (Sir John Megaw) and the Malay states (Dr. William Fletcher), encountered a vast array of “tropical fevers” [38,42,43,44]. Although endemic typhus-like illnesses were reported from various countries, little attention was shown to the sporadic typhus fevers (as compared with the epidemic typhus fevers). Working in India, Sir John Megaw, United Kingdom medical adviser to the India Office, responded to the “storm” of names for these diseases and the confusion of vectors, agents, and geographical regions by proposing in 1924 a new classification based on the vectors of different typhus forms (Figure 1) [44]. He distinguished between “epidemic” and “non-epidemic” typhus, and the old-world understanding of “typhus and unclassed fevers” was transformed into “the typhus group of fevers” [44]. Initially, this included the subgroups louse typhus, “tick-“, “mite-“, and “flea-typhus” [45], but with new cases of typhus in India, a new entity of “unknown vector-transmitted typhus” was added to the scheme in 1934 [46].

Due to the impressive diversity of vectors, animal reservoirs, and different geographical settings, a new era of confusion arose. In 1924, in the midst of this confusion, the term “tropical typhus” was coined in Malaya by William Fletcher. He states: *“… We call this variant ‘Tropical Typhus’ because it appears to be more common in the tropics than the epidemic form. It is necessary to distinguish it by some name—to call it simply ‘typhus’ is to mislead and alarm the public who, though they may be quite ignorant of everything else about typhus know that it’s highly infectious and may spread like a wildfire*.” [47]. Fletcher’s reports were very important in attracting the attention of the medical world to these sporadic fevers [42,48]. The fog dispersed as work was reported that allowed the “tropical typhus” of Fletcher to be separated into at least two entities.

Murine typhus (whose agent is now accepted as *Rickettsia typhi,* and whose vectors are fleas) was ultimately recognized as a separate disease from the epidemic typhus by three groups working independently: Fletcher in Malaya [38]; Hone, a public health doctor, in Adelaide, Australia [39]; and Maxcy and Havens, working in Montgomery, Alabama [49]. From 1931, true typhus was regarded as consisting of two distinct types: the classical European epidemic disease, associated with lice, and murine typhus, characterized independently by Maxcy [50] and Fletcher [38] in 1926, associated with fleas, and a natural reservoir in rats.

After the independent but simultaneous differentiation in the mid-1920s of murine typhus from epidemic typhus by Maxcy and from scrub or “rural” typhus by Fletcher in Malaysia, little research was performed, other than ongoing epidemiological surveillance. The main focus had shifted back to immunological studies in epidemic typhus. The major military consequences and the impact of rickettsial diseases during WWII, including their impact on troops in the AAP, have been well summarized [3,51,52,53]. Murine typhus occurred as sporadic cases in troops. The incidence of murine typhus actually exceeded that of epidemic typhus in the US Army in WWII [54]. There were essentially no cases of murine typhus recorded during the Korean conflict [8]. It was not generally considered a serious problem during the Vietnam conflict, but it was reported to be second only to malaria as a cause of fevers of unknown origin (FUO) in American personnel on bases and in cantonments [55].

Scrub typhus was a major infectious disease, which dominated WWII personnel in Asia. In some areas, it was second only to malaria in impact [51]. Its impact on immunologically naive Allied troops between 1942 and 1945 resulted in 16,000 cases and 639 deaths (4.0%) [52], as well as an estimated 20,000 cases in Japanese troops [2]. By the end of the war, scrub typhus was responsible for thousands of man-days lost and an estimated 7300 cases and 331 deaths (4.5%) in US troops [52]. Unlike the fatality rates of troops during WWII, scrub typhus caused no known American deaths during the Vietnam conflict [56]. However, serological data from reports for 1969 of the 9th Medical Laboratory, located in Saigon, shows that scrub typhus was the primary cause of fevers of unknown origin (18%), followed by amebiasis (17%) and murine typhus (15%).

It is impressive how the impact of scrub typhus affected research. This research expanded knowledge of the distribution of focal areas in the AAP and provided laboratory confirmation of the disease in these areas. Contributions in vector biology included the confirmation of natural infection in larval *Trombicula akamushi* [57], the first proof of natural infection in *T. deliensis* [58], and unequivocal evidence of transovarial and transstadial transmission of the agent through various stages of *T. deliensis* [51]. Evidence for the close taxonomic relationship of these two vector species was shown, and a significant increase in recognition of potential arthropod vectors and rodents as naturally infected mite hosts was described, as well as their environmental and geographic distributions [59].

Diagnostic improvements included refinement of laboratory techniques, discussed below, leading to the important discovery of antigenic differences between strains of *Rickettsia tsutsugamushi* (later changed to *Orientia tsutsugamushi*)*,* the agent of scrub typhus [60]. Preventive measures were improved, including environmental control, and development of better acaricides for personal protection by treatment of clothing. Most importantly, WWII greatly intensified the search for effective vaccines and for therapeutic drugs.

Thus, from an ill-defined mixture of fevers, the typhus fevers became clearly defined, including epidemic and murine typhus, with agents *R. prowazekii* and *R. typhi*, and scrub typhus and its agent *O. tsutsugamushi* representing the overwhelming proportion of cases in the AAP geographic region.

## 3. Evolution of Scrub Typhus and Rickettsial Diagnostics

The capability to diagnose rickettsial diseases has evolved dramatically since the early serendipitous discovery of the Weil–Felix agglutinations in 1916 for typhus, and in 1924–1925 for scrub typhus [61]. In the past twenty years, new serological assays, DNA sequence analysis, the polymerase chain reaction, and recombinant protein technology have greatly advanced and facilitated those early capabilities [5,62]. Here, we will briefly summarize early tests and modern advances focusing on tests developed or used in the AAP.

### 3.1. Weil–Felix Test

The Weil–Felix (WF) test is the oldest assay used for the diagnosis of rickettsial diseases [36,63,64]. The WF reaction is based on the cross-reaction of patient antibodies with SFGR, typhus group rickettsiae (TGR), and scrub typhus group orientiae (STGO) to antigenic epitopes of species of the genus *Proteus,* a member of the gamma proteobacteria. It is especially useful because it separates STGO from other rickettsiae. Although generally lacking sensitivity and specificity for diagnosis, this test has served its purpose well. Even with its weaknesses, the WF test is inexpensive, easy to perform, and readily accessible; which explains its continued use in certain regions of the AAP.

### 3.2. Complement Fixation Test

The complement fixation test (CF), also a test from the early rickettsial diagnostics era (1940s), could be used for both serodiagnosis and serotyping of the infecting strain of bacteria [65,66]. Guinea pig complement added to serial dilutions of the serum from patient or test animal binds to or “fixes” the complex, making it unavailable for lysis of added sheep red blood cells. Although endpoints are clear, the assay is quite tedious and time-consuming, and serum can be anticomplementary [67]. The amount of added complement must be precise. The assay test primarily measures specific immunoglobulin M (IgM). This test has mostly been displaced by newer more rapid technologies.

### 3.3. Indirect Immunofluorescence Assay

The indirect immunofluorescence assay (IFA) has been used for over 50 years to quantify anti-rickettsial antibodies including *O. tsutsugamushi-* and *R. typhi*-specific antibodies [68,69]. Each serum dilution is incubated on multiple distinct rickettsial antigen “dots” that are deposited on a single slide. The IFA test requires an expensive fluorescence microscope and a skilled technician to reproducibly read the endpoint. It can be used to serotype the infecting rickettsial strain [9]. IFA was used as the primary diagnostic procedure used in the papers by Stewart et al. [21], Selgado Lynn [22], and Yuhana et al. [28] in this special issue. In another paper [15], IFA provides part of the diagnostic regime utilized to study rickettsial infection in Bhutan. A test closely related to IFA, the indirect immunoperoxidase test (IIP) produces similar endpoint results but requires only a common light microscope to read the dilution endpoint [63,70]. In one paper of this special issue, IIP was used to examine cross-reactivity between *Rickettsia japonica* and *O. tsutsugamushi*, as well as the cross-reactivity observed between different serotypes of *O. tsutsugamushi* [14]. 

### 3.4. Rapid Diagnostic Tests

Commercial immunochromatographic rapid diagnostic tests (RDT) and immunodot assays produce rapid results, follow a simple protocol with no need for sophisticated electrical equipment, and are relatively inexpensive. They are highly attractive for point-of-care use in rural areas where the use of IFA may not be available. Moreover, the rapidity of these tests facilitates more rapid treatment, especially in rural areas with limited resources. The test(s) are based upon an enzyme-linked dot blot immunoassay, in which specific native antigens are immobilized on a membrane. Incubation of the membrane with patient sera allows for detection of antibodies to the antigen of interest on the membrane/dipstick. In the 1990s, many of these rapid dipstick/immunodot tests were developed through collaborations with the Department of Defense research laboratories and included assays for the detection of *R. typhi* and *O. tsutsugamushi* [71,72].

### 3.5. Rickettsial ELISA

The basic enzyme-linked immunosorbent assay (ELISA) is a technique to detect and quantify biologic substances such as peptides, proteins, lipopolysaccharides, antibodies, and hormones. In the simplest form, the ELISA is used to detect and quantify specific patient or animal antibodies against rickettsiae. Rickettsial antigens are attached to a solid surface, a 96-well plate or membrane, for example. As with most of the earlier serological assays, IFA, IIP, CF, dot blot assays, the ELISA was initially accomplished using native antigens derived from growing rickettsiae in embryonated hen eggs, or cell culture [73,74,75]. Dilutions of the patient antibody are incubated with the attached antigen to allow binding. Subsequently, an anti-antibody (anti-IgM or IgG, for example), which is linked to an enzyme, is incubated with the antigen–antibody complex on the plate or membrane. In the final step, a substance containing the enzyme’s substrate is added. Detection is accomplished by assessing the activity of the conjugated enzyme via incubation with the enzyme’s substrate to produce a detectable product, commonly a color change, that can be read by eye or intensity measured using a plate reader. The assay is easy to use, reproducible, and is more sensitive and specific than previous serological assays [64].

## 4. Diagnosis in the Era of Recombinant Proteins, DNA Sequencing and PCR (1987 to Present)

The first successful molecular cloning and gene expression of the rickettsial proteins involved the 110-kD and 56-kD proteins products of *O. tsutsugamushi*. Publication of the corresponding gene sequences introduced the field of rickettsiology to the molecular biology era and consequently to molecular diagnostics [76,77,78]. There was no longer a need for expensive biosafety level (BSL)-3 working standards to propagate live rickettsiae. The ability had been achieved to detect specific target rickettsial DNA in clinical samples. Detection of agent DNA could be done without animal inoculation or other cell culture isolation requirements [79,80].

### 4.1. Rickettsial ELISA Transformation: The Recombinants

With the introduction of molecular cloning, recombinant proteins based on the published sequences of rickettsial antigen genes were developed. Ching et al. developed a rapid immunochromatographic flow assay (RFA) for the detection of immunoglobulin M (IgM) and IgG antibodies to *O. tsutsugamushi* [81]. The RFA uses a truncated recombinant 56-kD protein from the Karp strain as the antigen. A sensitivity of 89% and specificity of 99% for IgM has been reported, showing that this approach is more sensitive than IFA, but limited by strain-specificity.

### 4.2. IgM ELISA

A most recent and promising point-of-care (POC) commercial serodiagnostic tool, the InBios Scrub Typhus Detect™ IgM ELISA, was evaluated in scrub typhus-endemic Northern Thailand [82] and Bangladesh [9]. The test uses recombinant p56-kD TSA56 of the *O. tsutsugamushi* strains Karp, Kato, Gilliam, and TA716 for scrub typhus IgM and IgG detection. When validated against the IFA, a sensitivity of 84% and specificity of 98% was noted in Northern Thailand, and in a subsequent evaluation, a sensitivity of 91.5% with specificity of 92.4% was reported for Bangladesh. Still, the InBios ELISA endpoints have not been firmly established [83]. The diagnostic accuracy of the InBios assay was investigated in a study of this special issue by Blacksell et al. [9]. InBios also markets a promising scrub typhus rapid test [84]. The Scrub Typhus Detect™ IgM ELISA System provided the primary diagnostic method used in the paper in the special issue by Gautum et al. [16].

### 4.3. Polymerase Chain Reaction (PCR) Diagnostics and Strain Typing

As noted above, PCR diagnostics were “off and running” in 1990 as gene sequences were being published. The initial PCR-based detection of *O. tsutsugamushi* targeted infected murine blood samples using primers derived from the just published *O. tsutsugamushi* 56-kD gene *tsa56* DNA sequences of Stover, et al. [77,80]. The first molecular diagnosis of typhus, also in 1990, was by Carl et al. [79], while *R. typhi* was detected in fleas by PCR in the same year [85]. Due to the “exceptional sensitivity” of the PCR, elaborate laboratory practices were developed to control “false positives.” PCR false positives are often the result of weak reactions from contamination and could confuse results. In addition to more stringent laboratory practices, newer, PCR-based assays soon followed, including the nested PCR, the loop-mediated isothermal amplification (LAMP) assays, and quantitative real-time PCR (qPCR) [64].

### 4.4. Gene Sequence Analysis

Associated with the products of PCR were various approaches to the characterization of strains for comparison with each other, comparison of strains within the different geographic regions, for example. The sensitive genotyping methods that became available included restriction fragment length polymorphism (RFLP), single-gene sequencing of highly variable genes, such as the 56-kD type-specific antigen (TSA56) of *O. tsutsugamushi*, multilocus sequence typing/analysis (MLST/MLSA), multispacer typing (MST), and whole-genome sequencing [64]. Gene sequence analysis forms an important tool for the comparison of standard strain of *O. tsutsugamushi* presented by Kelly et al. [12] in the special issue.

### 4.5. Quantitative PCR as an Emerging Diagnostic Tool

Quantitative real-time PCR, or qPCR [86,87], is now a well-established method for the detection, quantification, and typing of microbial agents in clinical diagnostics. It has been used as part of several studies in this special issue [15,88,89]. When applied correctly, qPCR is among the most straightforward measurement technique available for RNA and DNA quantification and is widely used in diagnostic and research applications. This powerful technique can provide precise and quantitative data, and the reverse transcriptase (RT)-qPCR for RNA has become well known worldwide as a rapid detection technology employed during the recent COVID-19 pandemic [90]. The main advantages of qPCR are that it provides fast and high-throughput detection and quantification of target nucleic acid sequences. The lower amplification time is promoted by the simultaneous amplification and visualization of newly formed nucleic acid amplification products. Moreover, qPCR is potentially safer than PCR and nucleic acid sequencing in terms of avoiding cross-contaminations because no further manipulation with samples is required after the amplification. However, unless strict guidelines are followed, including validation and data analysis procedures, the results can be ambiguous [91]. Procedures should be validated, as demonstrated in the paper by Reller and Dumler [88] in this special TMID issue, validating the use of qPCR in diagnosis of infections from forms of *Anaplasma* and *Ehrlichia*. Elementary protocol errors and inappropriate data analysis can occur, which can detract from the use of this powerful technique. Choice of source material is important. However, when using the specimen of choice, biopsy of rash and/or eschar, and strict adherence to guidelines, the sensitivity of qPCR assay approaches up to 100% [64]. Quantitative PCR also has a role in experimental analysis of rickettsial diseases, as illustrated by the paper of Naimi et al. in this issue [89], in which qPCR is used to assay differential bacterial load between male and female mice during infection by *Anaplasma phagocytophilum*.

### 4.6. How Diagnostic Tools were Utilized in Studies Reported in the Special Issue

In addition to some previous comments above concerning specific diagnostic tools, many of the papers included in this special issue make use of such tools in their studies. A number of papers utilized one or more of the diagnostic tools, often focusing their use to study the occurrence of rickettsial agents in particular geographic areas. Legendre and Macaluso [92] reviewed various procedures, including IFA, qPCR, and gene sequencing, that have contributed to insights on the biology of *Rickettsia felis*. Similarly, in their broad review of rickettsioses in Taiwan, Minahan et al. [93] indicate how a number of tools, IFA, PCR, RFLP analysis, qPCR, and gene sequencing, have all been used to study a number of different rickettsial disease agents occurring in the island nation. Hardstone Yoshimiru and Billeter [94] perform a similar review of many tools used in the study of North American rickettsioses. The paper by Luce-Federow et al. [4] discusses several of the diagnostic approaches reviewed above, but especially reviews how genomic advances in the study of scrub typhus might enhance diagnostics. Similarly, the review by Jiang and Richards [6] details how serology, IFA, ELISA, and DNA sequencing provide evidence that members of the genus *Orientia* extend beyond the AAP geographic region.

The possible combinations of diagnostic techniques that are being utilized in the analysis of scrub typhus are illustrated well in the study detailed by Naoi et al. [13], emphasizing their use in dealing with the severe complication of scrub typhus, hemophagocytic lymphohistiocytosis. Their Table 1 lists the various combinations of serological diagnostics that different patients received.

The choice of a single gold standard diagnostic tool remains unclear, due to the bi-phasic nature of early rickettsemia followed by a subsequent antibody response. The difficulty of diagnosis is illustrated in the paper by Salgado Lynn [22]. Reporting a case study, PCR analysis of the patient proved negative, while IFA and Northern blot analysis were required to correctly diagnose the cause of fever.

## 5. Evolution of Prevention and Treatments; Antibiotics and Rickettsial Vaccines in the AAP

### 5.1. Antibiotic Treatment of Scrub Typhus

The elevated caseloads and mortality rates caused by the rickettsial diseases during WWII, particularly scrub typhus in the AAP, were mission-threatening. The end of the war reduced the general concern, but investigators continued research and control efforts. Before the age of effective antibiotic therapy, scrub typhus patients suffered mortality rates of around 6–10%, sometimes exceeding 50% [41,95]—often with protracted convalescence of up to 4 months. This soon gave rise to the intensive drug development programs, which led to discovery of effective treatments and preventatives. Details of the dramatically successful treatment trial using Chloromycetin™ (Park-Davis, Detroit, MI, USA) have been well documented [3,11,96]. The trials, conducted in Malaysia, resulted in the first successful antibiotic treatment of human scrub typhus (1947–1948) [96]. The generic form of the compound, chloramphenicol, is still used when rapid therapy is warranted, but it has drawbacks; it can cause blood dyscrasias in the recovering patient.

Further research produced the bacteriostatic protein synthesis inhibitor, tetracycline (introduced in 1953), which proved even more effective than chloramphenicol in treating scrub typhus. Tetracycline showed fewer side effects and became the primary treatment [97]. Later, an oral form of the antibiotic, doxycycline (Pfizer, Inc., New York, NY, USA), was approved and came into common use to treat the majority of rickettsioses [98]. Generally, the treatment regimen for adults is doxycycline (200 mg/day for 7–15 days) or chloramphenicol (2 g/day for 7–15 days). These two antibiotics can be used to treat both scrub typhus and murine typhus [99]. Children would require a weight-dependent divided dose [100].

The case for treatment failures in scrub typhus has gained much attention in recent years [101,102], and summarized descriptions of the evidence have been presented elsewhere in the special issue of TMID [4,11,93]. Thus, in cases of doxycycline failure, azithromycin, 500 mg/day for 3 days, may be indicated [103]. Due to the complex nature of obligate intracellular parasites, the question of antimicrobial resistance deserves further careful scrutiny, as discussed in a recent evaluation by Wangrangsimakul, et al. [104]—where the authors conclude that earlier studies might have been interpreted differently and do not support evidence of classical resistance.

### 5.2. Vaccines

Exhaustive efforts to develop an effective, long-lasting, and broadly protective scrub typhus vaccine for humans have not proven successful. Those efforts extend from the WWII era when two vaccines were developed; one by the British based upon the Karp strain, and a second by US researchers based upon the Volner strain. Both were evaluated in the field and were found to not be significantly better than controls in preventing disease or death [105,106]. Immediately following the WWII era, the scrub typhus vaccine development efforts utilized *O. tsutsugamushi* isolates that were transported from Malaya to Britain [12,107]. Efforts in vaccine production up to the present run the gamut of inactivated and live-attenuated microorganisms and include recombinant protein-derived vaccines as well as DNA-plasmid vaccines, which are currently under investigation [108,109]. An example of the approach to use recombinant proteins to produce a viable vaccine is shown in the paper by Evans et al. in this special issue [10]. Success has been problematic, and there are currently no licensed vaccines for the prevention of any of the rickettsioses.

## 6. Molecular Variation of Scrub Typhus and Murine Typhus

### 6.1. Use of Single Genes versus Multiple Locus Sequence Typing (MLST)

Molecular approaches to the study of rickettsia now routinely use DNA sequencing as the gold standard for diagnosis and identification of rickettsial agents. These procedures often utilize DNA sequences of specific highly variable genes, such as the 56-kD type-specific antigen (TSA56) of *O. tsutsugamushi* [77,80], or rickettsial genes such as *gltA*, encoding rickettsial citrate synthase [110], *rOmpA*, encoding spotted fever group (SFG) rickettsiae-specific 190-kD outer membrane protein A [111], and the 17 kD gene, encoding the *Rickettsia* genus-specific 17-kD outer membrane antigen [112], each of which is useful and reliable for the identification of rickettsiae [113]. Analyses using these genes have been able to subdivide some species into genotypic groups [114], or to differentiate species [115], or to better understand the diversity of forms, such as was done by Hardstone Yoshimizu in this special issue of TMID [94].

Within *O. tsutsugamushi*, genotypic groups, such as those identified using the *tsa56* gene sequence, may reflect the phylogenetic histories of the isolates being compared [114]. However, population genetic studies based upon a single locus must be interpreted with caution, even when the locus being examined shows substantial variation. A number of studies in *O. tsutsugamushi* have suggested that the phylogenetic histories of different genes may not reflect a unified evolutionary history of the genes within the genome [116,117,118,119,120,121,122].

It has been suggested that some unique aspects of the genome of *O. tsutsugamushi* may facilitate significant horizontal gene transfer between isolates that would be reflected by incompatible phylogenetic histories for different genes. The characteristic of the genome that would potentially be most important in promoting substantial horizontal transfer is the large proportion of the *O. tsutsugamushi* genome that is made up of repeated sequences [118]. The original report of a genome sequence from *O. tsutsugamushi*, the genome of the Boryong strain, suggested that the sequence represented "the most highly repeated bacterial genome sequenced to date” [123]. The genomes of all *O. tsutsugamushi* strains that have been determined contain a large number of copies of genes for components of conjugative type IV secretion systems, for signaling and host-cell interaction proteins, as well as more than 400 copies of transposases, 60 copies of phage integrases, and 70 copies of reverse transcriptases. These multi-copy sequences have been hypothesized to facilitate intragenomic recombination. There is also the possibility that additional acceleration of strain differentiation may be associated with population bottlenecks that are likely to be associated with the intracellular nature and transmission patterns of rickettsial bacteria [124,125,126,127,128].

We are still at the early stages of understanding how *O. tsutsugamushi* evolves. Much remains to be determined concerning the population genetic dynamics of *O. tsutsugamushi*, and the comparison of those dynamics with patterns occurring in other rickettsiae that contain far fewer repeated components in their genomes. Studies of gene differentiation for multiple genes has been limited to a few studies of isolates from Southeast Asia [117,120,121,122] and single studies from Japan [119] and Korea [129].

In contrast, isolates that have been studied as part of genomic comparisons have been collected from a large part of the range of *O. tsutsugamushi* in the AAP. The most comprehensive analyses of *O. tsutsugamushi* phylogenetics were presented by Batty et al. [116] and Fleshman et al. [118]. Batty et al. identified a core set of 657 genes in eight isolates, including the prototype strains Gilliam, Karp, and Kato [12]. Some differences were found between the phylogeny from their merged dataset and individual phylogenies from the *tsa56* gene, the 47-kD *htraA* gene, and a seven-gene MLST panel. Differences between single-gene phylogenies and multigene phylogenies within the genome of a single species are expected to occur; the significance of this is more difficult to evaluate [130,131,132].

Horizontal gene transfer has been invoked to explain differences between gene trees within *O. tsutsugamushi* [116,118]. However, the process of incomplete lineage sorting may explain some, or even a substantial fraction, of these inconsistencies [133]. Much work remains to be done examining and comparing the distribution of different phylogenetic gene patterns to better understand the population dynamics of the agent of scrub typhus.

### 6.2. Genome Evolution within Rickettsial Species

The molecular revolution has raised several questions concerning the nature of evolution within the genome of *O. tsutsugamushi*. The genome sequences of isolates of *O. tsutsugamushi* reveal a completely different picture of genome change from that seen for genomes from *R. prowazekii* or *R. typhi*. In *Rickettsia*, gene order is maintained over large parts of the genus. Gene order is reasonably similar in various spotted fever group species. Comparisons between typhus group species and SFG forms show slightly more structural differences, primarily because a larger number of putative genes exist in the Typhus Group (TG) species, but most of the genome shows gene order preserved, although a few large inversions exist. For rickettsial forms within the AAP, a revealing difference between genome changes in *Rickettsia* and in *Orientia* can be seen when *R. typhi* genomes and *O. tsutsugamushi* genomes are examined. Genome sequences are available for five isolates of *R. typhi* (Wilmington [134], B9991CWPP, Ethiopia, TM2540, and TH1527). Three of these isolates, B9991CWPP, TH1527, and TM2540, were isolated within the AAP. Comparison of *R. typhi* isolates indicates that they all are co-linear in gene composition and order. In contrast, the order of genes within genomes of different isolates of *O. tsutsugamushi* is substantially different [116,118]. Only relatively small segments of the genome preserve gene co-linearity between isolates. There is strong evidence for the importance of transposable elements, which would facilitate recombination, resulting in scrambling the gene order within the genome. Much work remains to be done to further understand how genome structure affects disease in scrub typhus.

### 6.3. Geographic Patterns of Genome Differentiation

The overall mosaic pattern of genomes and the degree of gene differentiation between *O. tsutsugamushi* isolates remains incompletely understood. The degree of genetic differentiation between isolates is often limited to the context of single gene differences, or phylogenetic comparisons. Population genetics, including aspects of population size or the frequency of genetic “bottlenecks” will greatly alter the level of genetic variation that occurs within the “population” of a rickettsial species. With the availability of complete or nearly complete genome sequences, comparisons can be made at the level of genetic variation in different species. In the AAP, the appropriate comparison would be between the levels of variation occurring in *R. typhi* compared with the level of variation within *O. tsutsugamushi*.

Three of the five isolates of *R. typhi* for which genome sequences have been determined were isolated within the AAP: B9991CWPP, TH1527, and TM2540. Despite a worldwide distribution of isolates, pairwise comparisons between isolates of *R. typhi* indicate that they are extremely similar. Sequence conservation between the genomes of the five *R. typhi* isolates that have been collected from North America and Asia average 0.9998 over the entire ~1,112,400 bp long genome sequence, with differences primarily occurring because of in/del changes in noncoding simple repeats. In summary, *R. typhi* is essentially homogenous worldwide.

This is significantly different from the situation found for *O. tsutsugamushi* isolates that have been collected in the AAP. *O. tsutsugamushi* is very different from *R. typhi*. First, genome sizes are larger, almost twice as large as *R. typhi*, averaging 2.19 Mb among 13 genomes of *O. tsutsugamushi* for which total size has been carefully determined, compared with 1.11 Mb for *R. typhi* isolates. Much of the extra material comes in the form of duplicated sequences from a small number of elements [118,123]. The number of genes in the *O. tsutsugamushi* genome is larger because of these elements, with the *O. tsutsugamushi* isolates averaging almost three times as many coding sequences (2403 on average compared with 852). Gene order and gene number differs between isolates [116,118]. Pairwise comparisons of 178 genes found in sets of conserved gene blocks in the 13 "complete" sequences indicate levels of sequence similarity between 0.96 and 0.995. Pairwise comparisons for other genes are more difficult, in part because of assigning homology to loci being compared. In contrast, the *R. typhi* genomes can easily be compared over the entire length of the genome, with gene order conserved.

### 6.4. Geographical Patterns of Single-Gene Differentiation

Single-gene sequences in *O. tsutsugamushi* allow a different, but limited approach to the comparison of isolates. Much work remains to understand how the evolution of single genes corresponds to the genome evolution of isolates in *O. tsutsugamushi*. However, investigations of highly variable genes, especially the *tsa56* gene, do allow the examination of geographic variation within the species range of *O. tsutsugamushi*.

Variation of the *tsa56* gene is considerable, both within and between geographic areas of the AAP [114]. Diversity of sequences both within and between geographic locations has been observed, but the sequences of the *tsa56* gene may give us an insight only into the evolution of cell surface variation, and perhaps do not indicate how other genes would reflect evolution of the population. This caveat is inserted because it remains unclear whether the phylogenetic relationships among sequences of the *tsa56* gene would accurately reflect the phylogenetic relationship of other loci in the *O. tsutsugamushi* genome, or whether natural selection acting on the *tsa56* gene has altered the relationship among isolates in a different way compared with other loci in the genome. If natural selection acts specifically on the gene such as the *tsa56*, the phylogeny of the gene may not completely mirror the evolution of the organism, or the phylogeny of other genes in the genome.

Examination of the Table 1 shows clearly that different geographic areas are characterized by different frequencies of genetic types of the *tsa56* gene. The differentiation appears to be very significant. However, these numbers reflect the reporting of genetic sequences within the international DNA sequence databases, and do not necessarily reflect an epidemiologically appropriate sampling of isolates. Numbers in the table reflect a mixture of isolates from human cases and from mite vectors and rodent secondary hosts. Further, it is important to consider that some geographic regions in which scrub typhus is endemic are noted for the lack of information concerning genetic variability. These regions include Indonesia, Malaysia, and the Philippines and the large islands (New Guinea, Borneo, etc.) of the southwestern Pacific region. There is also minimal information concerning Myanmar, Pakistan, and associated regions at the western edge of the range of scrub typhus, and from northern Australia.

The *tsa56* gene has been used to study variation in *O. tsutsugamushi* carried by both mite vectors and in the rodent populations that represent the normal host for the mites. Evidence exists that different species of trombiculid mites may act as hosts to different genetic lineages of bacteria, and that different rodent hosts may also be nonrandomly associated with genetic lineages of *O. tsutsugamushi* [135].

### 6.5. Transmission Factors That Affect Geographic Gene Differentiation

The nature of transmission by vectors for scrub typhus may also contribute to the differences that are observed between *O. tsutsugamushi* and the two species in the typhus group of *Rickettsia*. Vertical transmission may be different for *O. tsutsugamushi* compared with the typhus group rickettsiae [125,126,127,136,137,138]. This may involve differences in both transstadial and transovarial transmission, and could contribute to the possibility of genetic exchange, and subsequent effects on genetic variability [126,127,128]. The ecology of scrub typhus is complex and still not completely understood [139]. It is known that multiple rodent species may act as host for the species of trombiculid mites that are the primary host for *orientiae* and the vector for spread of scrub typhus [139]. There is no strong evidence for any fidelity between genotypic strains of the bacteria to particular mite or rodent vector hosts. Some evidence exists of mites carrying more than a single *tsa56* genotype of *O. tsutsugamushi* [140]. Studies have reported multiple genotypes of *O. tsutsugamushi* within single rodent or mite hosts in nature [140]. Further, cases in which scrub typhus patients show evidence of infection by multiple genotypes of *O. tsutsugamushi* also exist [141,142,143].

Other ecological aspects of scrub typhus could also contribute to differentiation between intraspecific lineages of bacteria. The various mite vectors can demonstrate seasonal differences in their abundance [144,145,146,147]. This may be related to the seasonal occurrence of new scrub typhus infections [147,148,149,150,151] or differing annual activity patterns of *Leptotrombidium* species affecting the differentiation of bacterial lineages that they carry and transmit.

## 7. Where Are We in Our Study of Rickettsiology

Ultimately, continued research efforts are required for a better understanding about scrub typhus; currently, the focus should be on addressing entomological aspects in transmission, dissecting host–pathogen interactions in a clinical disease severity context, and in the development of point-of-care diagnostics and an effective vaccine.

Entomology: The recent discovery of UV-based autofluorescent microscopy in morphotyping *Leptotrombdium* mites has enabled for the first time paired-matched morpho- and genotyping, which should enable broader characterization of mites globally and their endosymbionts (and discovery of vertically transmitted pathogens), the molecular epidemiology of mites transmitting scrub typhus, as well as an improved understanding of adult mite stages [152].

Host–pathogen interactions: The availability of whole-genome sequencing provides a good platform to determine the virulence mechanisms of orientiae, as host-mediated pathogenic mechanisms and mechanisms of tissue injury remain poorly understood—ideally, these virulence factors should contribute towards developing a disease severity score/prediction based on clinical features and pathophysiological markers [153].

Diagnostics: A lot of effort has gone into improving the notoriously difficult diagnostics, but a universally useful point-of-care tool remains elusive. Ideally, this should comprise an antigen/nucleic acid-based component with an antibody detection step. Coupling this with the use of noninvasive or less–invasive sample specimens, such as sampling eschars or saliva, would enable earlier diagnosis, broader epidemiological coverage globally, and contribute substantially to improved awareness and better management of these easily treatable diseases [154]. Joint efforts in entomology and ecology with improved diagnostics will elucidate the true burden of disease and contribute to a better understanding, why the incidence of scrub typhus is rising in the AAP [95].

Vaccines: While extensive studies on features of the natural immune response were performed in the 1970–80s, this area has not received much attention lately—especially research with a focus on immune memory and clinically relevant correlates of protection is lacking. The ongoing genome-sequencing efforts, the availability of a characterized non-human primate model and first vaccine candidates conferring sterile immunity against high-dose homologous challenge with orientiae are promising prerequisites to progress this work [155].

AAP rickettsioses are a fascinating array of clinically relevant diseases—these endemic and underappreciated diseases are associated with a large burden of disease globally, and to date, there are still no licensed vaccines, or vector control efforts in place. Despite increasing awareness in endemic regions, the public health burden and global distribution of scrub typhus remains poorly understood. Opportunities are vast for the next generation of clinicians and scientists, there is still a lot to do.

## Figures and Tables

**Table 1:** Distribution among various locations of the *tsa56* gene sequences of *O. tsutsugamushi* deposited in the DNA databases, as placed into genotypic classes defined by Kelly et al. (2009) [111].

Genotype	Cambodia	India	Japan	Korea	Laos	PRC	Taiwan	Thailand	Viet Nam
Karp or Karp related	11	8	2	4	5	6	40	50	19
Jp-1 or Jp-2	2	-	48	6	-	3	6	4	1
Saitama	-	-	7	8	-	-	4	-	-
Kuroki	-	-	6	214	-	-	2	-	-
Gilliam	1	1	-	1	-	4	1	-	-
TA763	1	2	-	-	-	-	30	19	2
Kawasaki	-	-	7	58	-	91	2	-	-
JG	8	2	10	7	6	2	4	3	-
JG-v	-	4	1	-	-	-	27	22	3
Kato	4	-	-	2	-	-	10	1	-
Kato-v	2	24	-	-	-	1	16	7	-
Shimokoshi	-	-	9	-	-	1	-	-	-
Divergent	-	-	8	-	-	5	-	-	-
									
Total	29	41	98	300	11	113	142	106	25

## Supplementary Materials

The following are available online at https://www.mdpi.com/2414-6366/5/4/165/s1, Table S1: Historical timeline of the study of typhus.
