# Supplementary material for: A Brief History of the Major Rickettsioses in the Asia–Australia–Pacific Region: A Capstone Review for the Special Issue of TMID
Source: Trop Med Infect Dis. 2020 Oct 27;5(4):165. doi: 10.3390/tropicalmed5040165 (PMC7709643; doi:10.3390/tropicalmed5040165)
Supplement: Supplementary file 1 [file tropicalmed-05-00165-s001.pdf]

Table S1. Historical Chronology of Milestones - the Gradual Dissection of the "Typhus" Entity.

Year	Important findings	Comments	Ref.
460 BC	Hippocrates defines typhus - as "fever with a confused state of the intellect - a tendency to stupor".		[1]
430 BC	Thucydides and Hippocrates first describe epidemic typhus cases.		[2]
313 BC	First clinical accounts of *Tsutsugamushi disease* in the "*Zhouhofang*", a Chinese clinical manual.	Description of regional vector mites, no linkage to transmission	[3]
15th century	Epidemic typhus and endemic typhus (*Tabardillo*) rages in Europe.	No distinction made between different forms of typhus	[4]
1485–1551	The five epidemics known as the 'English Sweat' occur in the United Kingdom.	Probably relapsing fever (*Borrelia recurrentis*)	[5, 6]
1546	Girolamo Fracastoro–Fracastorius (1478−1553) differentiates typhus from plague in *De contagione et contagiosis morbis.*		[7-9]
Early 1700s	Typhus = Typhoid and Typhus and 'Relapsing Fever'	Era of differential diagnostic confusion!	[10–13]
1750	John Huxham made the first distinction between epidemic typhus and typhoid fever in the United Kingdom.	Typhus = ''slow nervous'' fever Typhoid = ''putrid malignant'' fever	[10]
1760	Boisier de Sauvages in Montpellier creates the clinical term "*Typhus exanthematique*" for epidemic typhus.	Based on clinical appearance of characteristic skin rash	[14]
1762	James Lind (1716−1794) promotes hygienic measures during typhus outbreaks to reduce mortality.	Limes for prevention of scorbut.	[8,9]
1810	Hakuju Hashimoto first described a *tsutsuga* (disease, harm, noxious) resembling typhus, in the Niigata prefecture, Japan.	The basis of the name '*Tsutsugamushi* disease'	[15]
1812–1813	Napoleon suffers the greatest loss of troops in eastern Europe to epidemic typhus (and Trench Fever).		[16,17]
1837	William W. Gerhard (1809−72), first distinguished enteric fevers (i.e. typhoid) from rickettsial fevers (i.e. typhus).	Post-mortem hyperplastic nodules in Peyer's patches	[11, 18]
1843	Craigie and Hendersen differentiate relapsing fevers clinically and pathologically from (epidemic) typhus	Post-mortem observations in autopsies	[12, 19]

1858	Charles Murchison epidemiologically emphasizes the contagious nature of typhus – association of epidemics with poor hygienic standards		[20]
1878	First account of *Tsutsugamushi* disease from Japan to be published in Europe by Theobald Palm.	Reported as Shima-mushi, or Island insect disease	[21]
1898	Brill Disease-an outbreak of atypical typhus, what Brill called abortive-typhoid in New York		[22,23]
1906	Howard T. Ricketts and Russel M. Wilder discover the causative agent and transmission vector (wood tick) of Rocky Mountain spotted fever (RMSF).		[24]
1908	Schüffner describes "Pseudo-typhoid" in Sumatra (later revealed to be *Tsutsugamushi* disease).	Suspected mite transmission	[25]
1909	Charles J.H. Nicolle discovers the transmission of epidemic typhus by the human body louse.	Nobel Prize in 1928	[26,27]
1910	Howard T. Ricketts first discovers rickettsiae in the blood of epidemic typhus patients in Mexico ("*Tabardillo*"), together with Russel M. Wilder.	Ricketts dies of epidemic typhus in Mexico 1910	[28−30]
1916	Rocha Lima who officially first described epidemic typhus rickettsiae as intracellular microorganisms.	*R. prowazekii* - named after Ricketts and Prowazek	[31]
1910	Smithson describes a "sporadic" form of typhus, in Australia.	Later revealed as scrub typhus or *Tsutsugamushi* disease	[32]
1910	Conor and Bruch describe "Fievre Boutonneuse" in Tunis.	Later revealed as Mediterranean Spotted Fever (MSF)	[33]
1911	McNaught describes "Para-typhoid" in South Africa, with suspected tick transmission.	Later revealed to be two forms; MSF and African Tick Typhus	[34,35]
1913	McKenchie describes a typhus-like illness in the Kumaon Region, N-India (Himalayan foothills).	unpublished, reviewed by Megaw 1921–"Megaw's tick bite" in 1916	[36]
1916	Weil and Felix first describe the diagnostic serum agglutination for typhus. Based on *Proteus vulgaris strain* called " *Proteus* X 19."	Diagnostic breakthrough	[37]
1922	Hone describes a new form of typhus in Australia (OX19 positive)	Later revealed as *R. honei*, a spotted fever group rickettsia	[38]

1923	Maxcy and Havens describe murine typhus as an endemic form of sporadic typhus.	Hypothesis that fleas are vectors	[39]
1924	Kingsbury visits Malaya and brings a new strain *Proteus mirabilis* OXK	OXK allows separation of "urban" and "rural" typhus	[40]
1924	Fletcher distinguishes scrub from murine typhus.	Based on the Weil-Felix test using *Proteus* OX19 and OXK	[41]
1926	Fletcher and Lesslar describe co-existance of *Tsutsugamushi* disease and scrub typhus in Malaya.		[42,43]
1926	Fletcher and Lesslar create the term 'tropical typhus'.	Defined as non-contagious or "sporadic" typhus-like fevers.	[40]
1928	Scrub typhus is *Tsutsugamushi* disease.	Based on entomological, cross-immunity and clinical criteria.	[41-45]
1930	Nagayo demonstrates *Rickettsia orientalis* for the first time.	Localization in the Descement's membrane of rabbit eyes.	[46]
1931	Dyer proves Maxcy right–murine typhus is transmitted by rat fleas.	Dyer isolates *Rickettsia* from rat fleas (*Xenopsylla cheopis*)	[47]
1945	"Operation Tyburn"–first mass-production of scrub typhus vaccine.	Based on New Guinea strain (Karp), did not work in Malaya.	[48−51]
1947	Discovery of Chloromycetin, a derivative from Actinomyces bacteria	Cured chick embryos infected with rickettsiae	[52]
1948	Smadel demonstrates chloramphenicol as an effective chemotherapy for scrub typhus.	Volunteer studies with naturally infected chigger inoculation	[52,53]
1959−1975	Vietnam Conflict–Scrub typhus is the leading cause of fevers of unknown origin (FUO).		[54]
1997	Epidemic typhus re-emerged dramatically in Burundi.	Epidemic affects approx. 100,000 refugees of Rwanda.	[55,56]

References

1. Lloyd, G.E.R., Chadwick J, Mann WN; *Hippocratic Writings;* Repr. ed.; Penguin Books: London, **1983,** 380 p.
2. Weiss K. The role of rickettsioses in history; In: *Biology of rickettsial diseases. Volume 1.* Edited by Walker D.H. , Boca Raton, FL, CRC Press, **1988,** 1-14.
3. Kawamura Jr, A., Tanaka H, Tamura A; *Tsutsugamushi Disease.* Tokyo University Press: Tokyo, **1995,** 362 pp.
4. Harden, V.A. Typhus, epidemic. *The Cambridge World History of Human Disease* **1993,** 1080-1084, DOI:10.1017/CHOL9780521332866.213.
5. Caius, J.: *A Boke Or Counseill Against the Disease Called the Sweate (1552);* Scholars' facsimiles & reprints: New York, **1937,** 1 p. of leaves, xix p., facsim.
6. MacArthur, W. Famine fevers in England and Ireland. *Ulster Med J* **1948,** 17, 28-33.
7. Fracastoro, G.: *Hieronymi Fracastorii De Contagione Et Contagiosis Morbis Et Eorum Curatione: Libri III (1546) [Translation and Notes.];* Putnam: New York, **1930,** 356 p.
8. Prinzing, F.: *Epidemics Resulting from Wars;* Oxford: Clarendon Press: London:, **1916,** xii + 340 + 6 p. appendix.
9. Lind, J.: *An Essay on the most Effectual Means of Preserving the Health of Seamen: In the Royal Navy.* D. Wilson: Lindon, **1762,** xxiv + 143 + 6 p addendum.
10. Huxham, J.: *An Essay on Fevers and their various Kinds (etc.) ;* 2nd ed.; Austen: Lomdon, **1750,** XV + 288 p.
11. Gerhard, W.W. ART. I. on the typhus fever, which occurred at Philadelphia in the spring and summer of 1836; illustrated by clinical observations at the Philadelphia hospital; showing the distinction between this form of disease and dothinenteritis or the typhoid fever with alteration of the follicles of the small intestine. *The American Journal of the Medical Sciences* **1837,** 19, 288-315.
12. Henderson, W. On some of the characters which distinguish the fever at present epidemic from typhus fever. *Edinburgh Med.Surg.J* **1844,** 61, 201-225.
13. Jenner, W.: *On the Identity Or Non-Identity of Typhoid and Typhus Fevers;* John Churchill: London, **1850,** vii + 102 p.
14. Hansen, W.; Freney, J.; Le typhus épidémique, sa transmission et la découverte de l'agent étiologique. *Lyon Pharmaceutique* **1996,** 3, 130-138.
15. Shao, P. You Xing and Hakuju Hashimoto - pioneers of epidemic doctrine in the history of diseases in China and Japan (in Japanese) . *Journal of the Japanese Society for the History of Medicine (Nihon Ishigaku Zasshi)* **2001,** 47, 604-605.
16. Peterson, R.K.D. Insects, disease, and military history. *Am Entomol* **1995,** 41, 147-161, DOI:10.1093/ae/41.3.147.
17. Raoult, D.; Dutour, O.; Houhamdi, L.; Jankauskas, R.; Fournier, P.E.; Ardagna, Y.; Drancourt, M.; Signoli, M.; La, V.D.; Macia, Y.;Aboudharam, R.; Evidence for louse-transmitted diseases in soldiers of Napoleon's grand army in Vilnius. *J Infect Dis* **2006,** 193, 112-120, DOI:10.1086/498534.
18. JAMA Editorial. W. W. Gerhard, typhoid vs. typhus fever. *JAMA : The Journal of the American Medical Association* **1962,** 181, 154-155, DOI:10.1001/jama.1962.03050280084012.

19. Craigie, D. Notice of a febrile disorder which has prevailed at Edinburgh during the summer of 1843. *Edinburgh Medical and Surgical Journal* **1843,** 60, 410.
20. Murchison, C. Typhus fever. *Br Med J* **1858,** 1, 994.
21. Palm, T.A. Some account of a disease called" shima-mushi," or" island-insect disease," by the natives of Japan; peculiar, it is believed, to that country, and hitherto not described. *Edinburgh Medical Journal* **1878,** 24, 128.
22. Brill, N.E. An acute infectious disease of unknown origin - A clinical study based on 221 cases. *Am J Med Sci* **1910,** 139, 484-502.
23. Brill, N.E. Pathological and experimental data derived from a further study of an acute infectious disease of unknown origin. *The American Journal of the Medical Sciences (1827-1924)* **1911,** 142, 196-218.
24. Ricketts, H.T. The transmission of rocky mountain spotted fever by the bite of the wood-tick (*Dermacentor occidentalis*). *Journal of the American Medical Association* **1906,** XLVII, 358, DOI:10.1001/jama.1906.25210050042002j.
25. Schüffner, W.; Wachsmuth, M.; Uber eine typhusartige erkrankung (pseudotyphus von deli). *Z Klin Med* **1910,** 71, 133-156.
26. Gross, L. How Charles Nicolle of the Pasteur institute discovered that epidemic typhus is transmitted by lice: Reminiscences from my years at the Pasteur institute in Paris. *Proc Natl Acad Sci U S A* **1996,** 93, 10539.
27. Nicolle, C.; Comte, C.;Conseil, E.; Transmission expérimentale du typhus exanthématique par le pou du corps. *C. R. Acad. Sci.* **1909,** 149, 486-489.
28. Ricketts, H.T.; Wilder, R.M.; Further investigations regarding the etiology of tabardillo, Mexican typhus fever. *J Am Med Assoc* **1910,** 55, 309-311, DOI:10.1001/jama.1910.04330040045015.
29. Ricketts, H.T.; Wilder, R.M.; The relation of typhus fever (tabardillo) to rocky mountain spotted fever. *Arch Intern Med* **1910,** 5, 361-370.
30. Ricketts, H.T.; Wilder, R.M.; The typhus fever of mexico (tarbadillo) preliminary observations. *J Am Med Assoc* **1910,** 54, 463-467.
31. da Rocha-Lima, H. Zur aetiologie des fleckfiebers. *DMW-Deutsche Medizinische Wochenschrift* **1916,** 42, 1353-1354.
32. Smithson, O. Mossman fever. *J.Trop.Med Hyg* **1910,** 13, 351-352.
33. Conor, A.; Brush, A.; Une fievre boutonneuse observee en Tunisie. *Bulletin De La Societe De Pathologie Exotiques Et Filiales* **1910,** 8, 492-496.
34. McNaught, J.G. A note on two cases of paratyphoid fever in which a new variety of paratyphoid bacillus was found in the blood. *BMJ Military Health* **1908,** 10, 171-174.
35. Troup, J.M.; Pijper, A.; Tick-bite fever in southern Africa. *Lancet* **1931,** .
36. Megaw, J. A typhus-like fever in India, possibly transmitted by ticks. *The Indian Medical Gazette* **1921,** 56, 361-371.
37. Weil, E.; Felix, E.; Zur serologischen diagnose des flekfiebers. *Wien Klin Wochenschr* **1916,** 29, 33-35.
38. Hone, F.S. A series of cases closely resembling typhus fever. *Med J Aust* **1922,** 1, 1-13.
39. Maxcy, K.F.; Havens, L.C.; A series of cases giving a positive Weil-Felix reaction. *Am J Trop Med Hyg* **1923,** s1-3, 495-507, DOI:10.4269/ajtmh.1923.s1-3.495.

40. Audy J. R.; Savoor S. R.; Typhus; In: *The Institute for Medical Research 1900-1950 by various authors; Studies from the Institute for Medical Research, no. 25.* Edited by Field J.W.; Green R.; Byron F. E.; , **1951,** .
41. Fletcher, W.; Lesslar, J.E.; Tropical typhus and Brill's disease. *J Trop Med Hyg* **1926,** 29, 374-378.
42. Fletcher, W.; Lesslar; J.E.;Lewthwaite, R.; The ætiology of the tsutsugamushi disease and tropical typhus in the federated Malay states: A preliminary note. part I. . *Trans R Soc Trop Med Hyg* **1928,** 22, 161-174, DOI:10.1016/S0035-9203(28)90008-5.
43. Fletcher, W.; Lesslar, J.E.;Lewthwaite, R.; The aetiology of the tsutsugamushi disease and tropical typhus in the federated Malay states. part II. *Trans R Soc Trop Med Hyg* **1929,** 23, 57-70, DOI:10.1016/S0035-9203(29)90849-X.
44. Audy, J.R.: *Red Mites and Typhus.* University of London, The Athlone Press: London, **1968,** x + 191.
45. Gater, B.A.R. Entomological investigations in relation to tropical typhus in Malaya. *Far Eastern Ass. of Trop. Med., Transac. 8th Cong., Siam* **1930,** 2, 132-141.
46. Nagayo, M.; Tamiya, T.; Mitamura, T.;Sato, K.; On the virus of tsutsugamushi disease and its demonstration by a new method. *Jikken Igaku Zasshi= Japanese Journal of Experimental Medicine* **1930,** 8, 309-318.
47. Dyer, R.E.; Ceder, E.T.; Lillie, R.D.; Rumreich, A.;Badger, L.F.; The experimental transmission of endemic typhus fever of the United States by the rat flea *Xenopsylla cheopis*. *Public Health Reports (1896-1970)* **1931,** 2481-2499.
48. Fulton, F.; Joyner, L.; Cultivation of rickettsia tsutsugamushi in lungs of rodents - preparation of a scrub-typhus vaccine. Lancet 1945, 249, 729-734.
49. Buckland, F.E.; Dudgeon, A.; Edward, D.G.; Henderson-Begg, A.; MacCallum, F.O.; Niven, J.; Rowlands, I.W.; Ende, M.; Bargmann, H.E.;Curtis, E.E.; Scrub-typhus vaccine: Large-scale production. Lancet 1945, 249, 734-737.
50. Chattopadhyay, S.; Jiang, J.; Chan, T.; Manetz, T.S.; Chao, C.; Ching, W.;Richards, A.L.; Scrub typhus vaccine candidate kp r56 induces humoral and cellular immune responses in cynomolgus monkeys. Infect Immun 2005, 73, 5039-5047, DOI: 10.4161/hv.3.3.4009.
51. Henderson-Begg, A.; Fulton, F.; The standardisation of a scrub typhus vaccine. *J Pathol Bacteriol* **1946,** 58, 381-389, DOI:10.1002/path.1700580308.
52. Ehrlich, J.; Bartz, Q.R.; Smith, R.M.; Joslyn, D.A.;Paul R. Burkholder; Chloromycetin, a new antibiotic from a soil actinomycete. *Science (Wash)* **1947,** 106, 417, DOI:10.1126/science.106.2757.417.
53. Smadel, J.E.; Jackson, E.B.; Chloromycetin, an antibiotic with chemotherapeutic activity in experimental rickettsial and viral infections. *Science (Wash)* **1947,** 106, 418-419, DOI:10.1126/science.106.2757.418.
54. Deller J. J. Fever of undetermined origin. In: *Internal Medicine in Vietnam.* Edited by Ognibene A.F., Barrett O. J.; , Wadhington, D.C., Office of the Surgeon General and Center of Military History United States Army, **1982,** 78-89.
55. Raoult, D.; Roux, V.; Ndihokubwayo, J.B.; Bise, G.; Baudon, D.; Martet, G.;Birtles, R.; Jail fever (epidemic typhus) outbreak in Burundi. *Emerg Infect Dis* **1997,** 3, 357-360, DOI:10.3201/eid0303.970313.

56. Raoult, D.; Ndihokubwayo, J.B.; Tissot-Dupont, H.; Roux, V.; Faugere, B.; Abegbinni, R.;Birtles, R.J.; Outbreak of epidemic typhus associated with trench fever in Burundi. *Lancet* **1998,** 352, 353-358, DOI:10.1016/S0140-6736(97)12433-3.
